# Moving system with action sport cameras: 3D kinematics of the walking and running in a large volume

**DOI:** 10.1371/journal.pone.0224182

**Published:** 2019-11-12

**Authors:** Gustavo R. D. Bernardina, Tony Monnet, Pietro Cerveri, Amanda P. Silvatti

**Affiliations:** 1 School of Physical Education, Physiotherapy and Occupational Therapy, Universidade Federal de Minas Gerais, Belo Horizonte, Minas Gerais, Brazil; 2 Department of Biomechanics and Robotics, PPRIME Institute, CNRS – University of Poitiers – ENSMA, UPR 3346, Poitiers, France; 3 Eletronics, Information and Bioengineering Department, Politecnico di Milano, Milano, Italy; 4 Department of Physical Education, Universidade Federal de Viçosa, Viçosa, Minas Gerais, Brazil; University of Pittsburgh, UNITED STATES

## Abstract

Traditionally, motion analysis in clinical laboratories using optoelectronic systems (MOCAP) is performed in acquisition volumes of limited size. Given the complexity and cost of MOCAP in larger volumes, action sports cameras (ASC) represent an alternative approach in which the cameras move along with the subject during the movement task. Thus, this study aims to compare ASC against a traditional MOCAP in the perspective of reconstructing walking and running movements in large spatial volumes, which extend over the common laboratory setup. The two systems, consisting of four cameras each, were closely mounted on a custom carrying structure endowed with wheels. Two different acquisition setups, namely steady and moving conditions, were taken into account. A devoted calibration procedure, using the same protocol for the two systems, enabled the reconstruction of surface markers, placed on voluntary subjects, during the two acquisition setups. The comparison was quantitatively expressed in terms of three-dimensional (3D) marker reconstruction and kinematic computation quality. The quality of the marker reconstruction quality was quantified by means of the mean absolute error (MAE) of inter-marker distance and two-stick angle. The kinematic computation quality was quantified by means of the measure of the knee angle reconstruction during walking and running trials. In order to evaluate the camera system and moving camera effects, we used a Wilcoxon rank sum test and a Kruskal Wallis test (post-hoc Tukey), respectively. The Spearman correlation coefficient (*ρ*) and the Wilcoxon rank sum test were applied to compare the kinematic data obtained by the two camera systems. We found small ASC MAE values (< 2.6mm and 1.3°), but they were significantly bigger than the MOCAP (< 0.7mm and 0.6°). However, for the human movement no significant differences were found between kinematic variables in walking and running acquisitions (p>0.05), and the motion patterns of the right-left knee angles between both systems were very similar (*ρ*>0.90, p<0.05). These results highlighted the promising results of a system that uses ASC based on the procedure of mobile cameras to follow the movement of the subject, allowing a less constrained movement in the direction in which the structure moves, compared to the traditional laboratory setup.

## Introduction

Three-dimensional (3D) kinematic analysis systems, using optoelectronic cameras and hardware /software processing units, are traditionally used in laboratory setup where they are able to reconstruct highly accurate 3D kinematic data (mean absolute error < 0.3 mm [1]). However, as the cameras are stationary, the resulting acquisition volume allows the study of cyclical movements featuring on average only 3 to 6 stride cycles [2,3]. While in principle the use of additional cameras, distributed over a larger spatial volume, can overcome this limitation, the significant increase in costs and the complexity of the resulting setting reduce the extent of this approach to real applications.

In order to address the demand of wider volume recordings, especially mandatory for cyclical motions, stationary equipment as treadmills and ergometers were proposed ([4]–treadmill; [5]–cycle ergometer; [6]–rowing ergometer). In walking, for example, it is shown that treadmill and overground movements are similar, but can presents significant differences for kinematic parameters are reported, although the magnitude of these differences can be small [4]. Nonetheless, because of restrictions to the allowed movement excursion, such tools only partially reproduce the real motion and the treadmill walking and running mechanics cannot be generalized to the overground condition [7,8].

Inertial sensors have been recently investigated for indoor and outdoor motion analysis, however poor accuracy results, compared to traditional optoelectronic systems [9,10,11] have discouraged their systematic use in both clinics and sport science applications. A paradigm change is represented by the use of cameras that follow the subject during the movement task. This setup requires that the cameras are placed on a rigid frame mounted on wheels. Interestingly, some studies in the literature, addressing this approach by means of traditional optoelectronic systems, reported accuracy results comparable to those provided by stationary motion capture systems [12,13]. However, the configuration is cumbersome as it requires that the video processing unit, connected to the cameras, be positioned on the mobile structure and the movement of this structure can be difficult. An interesting solution, which addresses both wiring and costs of the system, is represented by the use of wireless consumer video systems such as the action sport cameras (ASC). Recently, these cameras were assessed in comparison with a commercial motion capture system and, although they present lower precision and accuracy in the marker reconstruction, this did not affect the 3D kinematic calculations of the movements reconstructed were less affected [1]. Thus, from a perspective of using alternative systems to traditional optoelectronic devices that are expensive, it is necessary to further stud the feasibility of ASC in biomechanics applications.

Since 2012, our group has been investigating ASC to perform 3D kinematic evaluation for both laboratory and outdoor applications by addressing the role of calibration tools, image acquisition, image processing, camera setup and calibration and marker tracking [14,15,16,17,1]. Capitalizing on such earlier knowledge, in this work we performed a feasibility analysis of using ASC moving cameras to reconstruct 3D kinematics of the gait movements (walking and running) in a large spatial volume, in comparison with a traditional optoelectronic system. We hypothesized some differences in the marker reconstruction quality because of cameras mounted on the mobile structure. Nonetheless, we expected less difference in computation of the kinematic variables describing walking and running movements.

## Methods

### System setup

Four cameras ViconMX40 (MOCAP—Motion capture system—Vicon. Oxford Metrics Ltd. UK) and four Action Sport Cameras (ASC—GoPro. Hero3 +. Black Edition^®^ / USA) were available for the study and three experimental situations were considered: the reference, the steady structure and mobile structure setups, named setup #1, #2 and #3, respectively. For all of them, the same camera data were adopted (Table 1).

**Table 1:** Camera data.

**Type of camera**	**ASC**			**MOCAP**		
**Number of camera**	4			4		
**Image resolution (pixels)**	1280 x 720			2353 x 1728		
**Acquisition frequency (Hertz)**	120			120		
**Illumination**	Ring with 4 LEDs			Ring with 320 LEDs		
**Setups**	Reference [#1]	Steady structure [#2]	Mobile structure [#3]	Reference [#1]	Steady structure [#2]	Mobile structure [#3]

ASC = Action Sport Camera; MOCAP = Motion Capture System.

**Fig 1:**
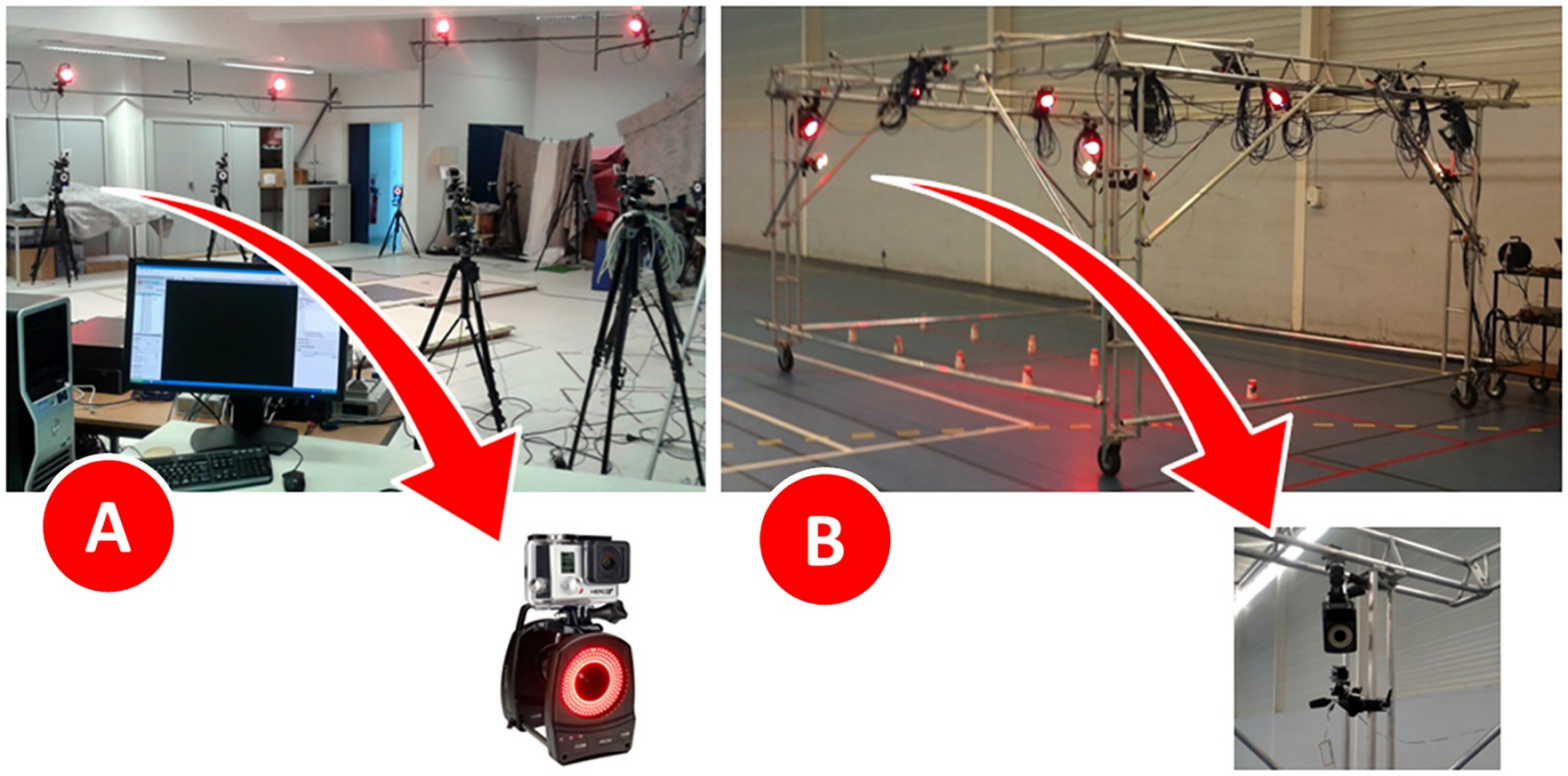
Cameras position. (A) Cameras position in the setup #1 (Reference). (B) Cameras position in the setup #2 (Steady structure) and setup #3 (Mobile structure, that involved the movement of the structure along a straight-line trajectory of 40 meters).

In the setup #1 (reference), the 3D accuracy results obtained through a traditional setup of a motion analysis laboratory, recently reported in [1], were adopted. The cameras were fixed in tripods with the ASC hanging in the MOCAP, to ensure a close proximity between camera pairs and were placed at the corners of the rectangle encircling the working volume (approximately 4.0 x 1.5 x 2.0 m, the typical size for gait analysis) (Fig 1A). More detailed information can be checked in [1].

In the setup #2 (steady structure) and #3 (mobile structure), a customized rigid structure mounted on wheels (4.4×4×2.5 m) was adopted to transport the cameras, positioned at a height of about 2.30 meters from the ground (Fig 1B). The data acquisitions were performed in these two different setups, one involving the steady location of the structure (working volume approximately 4.0 x 2.0 x 2.0 m) whereas in the second one the structure was moved along a straight-line trajectory of 40 meters on an indoor field for handball (working volume approximately 40.0 x 2.0 x 2.0 m). There is no still mechanism for automated movement of the structure, so it was moved by the researchers who conducted this work. The speed of movement of the structure followed the self-selected speed of the subject during the tasks.

The ASC were all synchronized by means of the GoPro Wi-Fi remote control. In the three experimental situations, to synchronize the data acquisition between the two acquisition systems and to define the initial frame of interest, a light signal was used.

### Camera calibration protocol

The cameras were calibrated before each trial and the MOCAP camera calibration protocol was adopted for both systems (Table 2). For the setup #1 (reference), this protocol required first the acquisition of wand, carrying five markers, which was moved within the working volume and second the acquisition of a calibration frame, located on the floor, according to the method described in [1]. For the MOCAP, the video acquisitions were automatically processed by proprietary software. For ASC, the captured videos of the same acquisitions were converted to AVI movie format and processed by a custom software environment developed in Matlab^®^ 2015a (Mathworks, Natick MS) to semi-automatically detect and extract markers from the images processing [14,1]. For setup #2 (steady structure), the ASC camera calibration was performed the same way as in the setup #1. For setup #3 (mobile structure), algorithms were developed to adapt the camera calibration of setup #1 computing the frame-by-frame roto-translation using three non-aligned reconstructed marker placed on the ground as the cameras moved. The roto-translation of the reference system happened during all the movements’ execution path. The marker reconstruction required the concurrent acquisition of forty-one markers put on the ground, along the length of the volume, in two parallel lines. In each line, the markers were positioned at a relative distance of 1 meter. Extended details about this procedure can be found in [13]. The optical distortion was taken into account adding one radial parameter into the camera model, since the ASC were set as a ‘narrow’ field of view.

**Table 2:** Summary of camera calibration protocol and systems evaluation.

Setups		#1	#2	#3
**Camera calibration protocol**	Calibration tool	MOCAP T-shape	MOCAP T-shape	MOCAP T-shape
	Marker size	14 mm	14 mm	14 mm
	Calibration data acquisition	MOCAP protocol	MOCAP protocol	Begon *et al*., 2009
**Experimental protocol**	3D reconstruction accuracy	Rigid bar test	Rigid bar test	Rigid bar test
	Human kinematic data	Walking	-	Walking and Running

mm = millimeters.

### Experimental protocol

#### 3D reconstruction accuracy

The 3D reconstruction accuracy evaluated the 3D markers reconstruction quality in relation to the system performance and to the different setups. We performed a rigid bar test in which were considered three non-aligned markers on the calibration frame (MOCAP T-shape wand tool). Two virtual segments, defined by the first and the second markers and the second and the third markers, represented two known inter-marker distances (D1: 160mm and D2: 240mm), respectively, and a known angle (α = 90°), according to manufacturing precision provided by MOCAP system.

The 3D reconstruction accuracy was tested in one trial for each experimental situation (Table 2). These tests involved the acquisition, by the two systems concurrently, of the frame moved by hand within the calibration volumes. It was captured approximately 10 seconds to setup #1, 20 seconds to setup #2 and 40 seconds to setup #3.

The accuracy indexes of interest were the mean error of the inter-marker distance (ME); the standard deviation error of the inter-marker distance (SD), and the mean absolute error of the distance (MAE). The same accuracy indexes were calculated for the angle shaped between the three markers.

#### Human kinematic data

We used the setup #1, a common gait lab configuration, and the setup #3, an out-of-lab condition with cameras in motion, to compare the human kinematic data obtained by the two systems concurrently (Table 2). The markers’ placement in one healthy subject (female, 32 years old, 1.7 m and 58.5 kg) was adapted according to Plug-In Gait Lower-Limb model. Fourteen retro-reflective markers (⊘: 14 mm) were placed on the following anatomical points of the both lower limbs: anterior and posterior superior iliac spines, femur greater trochanter, femur lateral epicondyle and lateral malleolus, 5th metatarsus and calcaneus. In the setup #1, three walking trials were acquired and in each trial one gait cycle for right and one gait cycle for left limb were selected. In the setup #3, the same subject performed one walking trial and one running trial through, approximately 40 meters, being possible to acquire 17 and 14 gait cycle, respectively. In both setups, all trials were performed to a self-selected speed with a natural cadence and were acquired concurrently by both systems. This study was approved by the Universidade Federal de Viçosa Ethics Committee (CAAE: 49426115.1.0000.5153).

The reconstructed 3D coordinates of the markers placed in the subject underwent pre-processing by means of a 15Hz cut-off Butterworth filter [18]. The following angular and linear parameters of the walking and running were calculated for both limbs: (a) right and left knee flexion-extension angle as a function of time; (b) maximum knee angle; (c) mean velocity, monitored by the mean point of the pelvis markers as a function of time; (d) stride lengths.

### Statistical analysis

In order to analyze the overall accuracy of the 3D reconstruction we used the MAE values (D1, D2 and the angle between the markers on the frame). All statistical analysis were performed in Matlab^®^ 2015a (Mathworks, Natick, MA) assuming statistical significance of *p*<0.05. The test to evaluate the data distribution (Lilliefors test) was applied to the absolute errors of the inter-marker distance value and of the angle shaped between the three markers of each experimental condition and revealed a non-normal distribution. Thus, the system performance was evaluated in each setup (#1, #2 and #3) applying the non-parametric tests (Wilcoxon rank sum) to compare the ASC and the MOCAP. To analyze the differences between the setups in each systems (ASC and MOCAP, separately), we applied the non-parametric test (Kruskal Wallis, *post-hoc* Tukey).

To evaluate the human kinematic data computed with the setup #3, we first compared the knee flexion-extension angle curves, obtained by both systems, ASC and MOCAP. Both for the walking and for the running, we applied the Spearman correlation coefficient (*ρ*) for each cycle, on both lower limbs, in order to analyze the relationship of the waveform pattern of the knee angular variation between the two systems. Second, we applied the Wilcoxon rank sum test to compared the values of the angular (maximum knee angle values of the right and left lower limb) and linear (stride length) parameters obtained in each system. For these comparisons, the effect size (r) was calculated, in which the values above of 0.8, between 0.79 and 0.5, between 0.49 and 0.2 and below 0.19 represented a large, medium, small and insignificant effect, respectively [19,20]. For the walking and running 17 and 14 gait cycles were considered, respectively. As only one trial of each task was performed, the mean velocity was presented as descriptive statistics.

The human kinematic data obtained in the setup #1 were presented in the result, but the statistical analysis can be verified in [1].

## Results

### 3D reconstruction accuracy

The results of the variables analyzed in the 3D reconstruction accuracy were presented in the Table 3.

**Table 3:** Mean errors (real value—Value obtained), standard deviations, mean absolute errors of the inter-marker distance 1 (160mm), distance 2 (240mm) and angle between markers (α = 90°).

Setup		#1			#2			#3		
		Mean Error	Standard Deviation	Mean Absolute Error	Mean Error	Standard Deviation	Mean Absolute Error	Mean Error	Standard Deviation	Mean Absolute Error
**D1 (mm)**	ASC	2.15	2.10	2.47	1.72	2.68	2.56	1.38	1.88	1.91
	MOCAP	-0.06	0.28	0.17	-.014	0.12	0.15	-0.03	0.96	0.30
	***p-*value**	-	-	<0.0001	-	-	<0.0001	-	-	<0.0001
**D2 (mm)**	ASC	-0.60	1.82	1.53	0.94	1.92	1.62	1.58	2.07	1.99
	MOCAP	-0.01	0.27	0.18	-0.49	0.09	0.49	-0.44	1.05	0.62
	***p-*value**	-	-	<0.0001	-	-	<0.0001	-	-	<0.0001
**Angle (°)**	ASC	-0.13	0.70	0.57	-1.02	0.90	1.17	-1.20	0.66	1.22
	MOCAP	0.46	0.12	0.47	-0.46	0.03	0.46	-0.45	0.40	0.51
	***p-*value**	-	-	0.118	-	-	<0.0001	-	-	<0.0001

D1 = inter-marker distance 1 (160mm); D2 = inter-marker distance 2 (240mm); mm = millimeters; ASC = Action Sport Camera; MOCAP = Motion Capture System; *p*-value = comparison of the MAE between ASC and MOCAP in each experimental situation, *p*<0.05, Wilcoxon rank sum test.

In agreement with previous findings in the literature [1], the MOCAP provided the better accuracy results (Table 3). The MAE of the inter-markers distance (D1 and D2) was significantly lower than the ASC in the setup #1, performed in laboratory with controlled environment. These differences were kept in both setup #2 and #3. As far as MAE of the angle is concerned, we found similar results, with exception of the setup #1, in which the values among the systems were not significantly different (Table 3).

In the ASC, the change in the setup did not affect the MAE related to the inter-marker distance (D1, setup #1 *vs* #2, *p* = 0.99; D2, setup #1 *vs* #2, *p* = 0.99), instead the MAE of the angles was affected, and worse angle values were obtained (setup #1 *vs* #2, *p*<0.0001). In setup #3, the MAE related to the inter-marker distance and angle was affected by the movement of the structure (setup #1 *vs* #3, *p*<0.0001 and setup #2 *vs* #3, *p*<0.0001).

In the MOCAP, the change in the setup affected negatively one of the MAE of the inter-marker distances (D2, setup #1 *vs* #2, *p*<0.0001) and maintained the MAE values of the other inter-marker distance (D1, setup #1 *vs* #2, *p* = 0.1975). Related to the MAE of the angles, we found similar results of the ASC (setup #1 *vs* #2, *p*<0.0001). Again, setup #3 affected negatively the MAE values of the inter-marker distances (D1, setup #1 *vs* #3, *p*<0.01 and setup #2 *vs* #3, *p*<0.01; D2, setup #1 *vs* #3, *p*<0.0001 and setup #2 *vs* #3, *p*<0.001) and angle (setup #1 *vs* #3, *p*<0.0001 and setup #2 *vs* #3, *p*<0.0001).

### Human kinematic data

**Fig 2:**
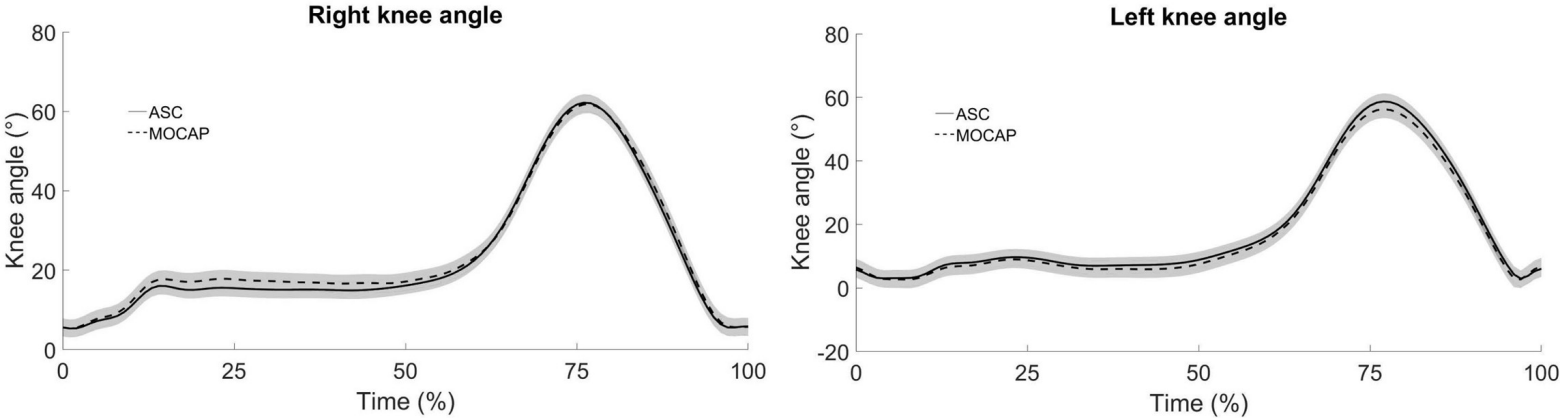
Mean curve of the angular variation, of the right and left knee, of 17 stride cycles in one walking trial (40 meters). Vicon (dashed line) and GoPro (continuous line).

**Fig 3:**
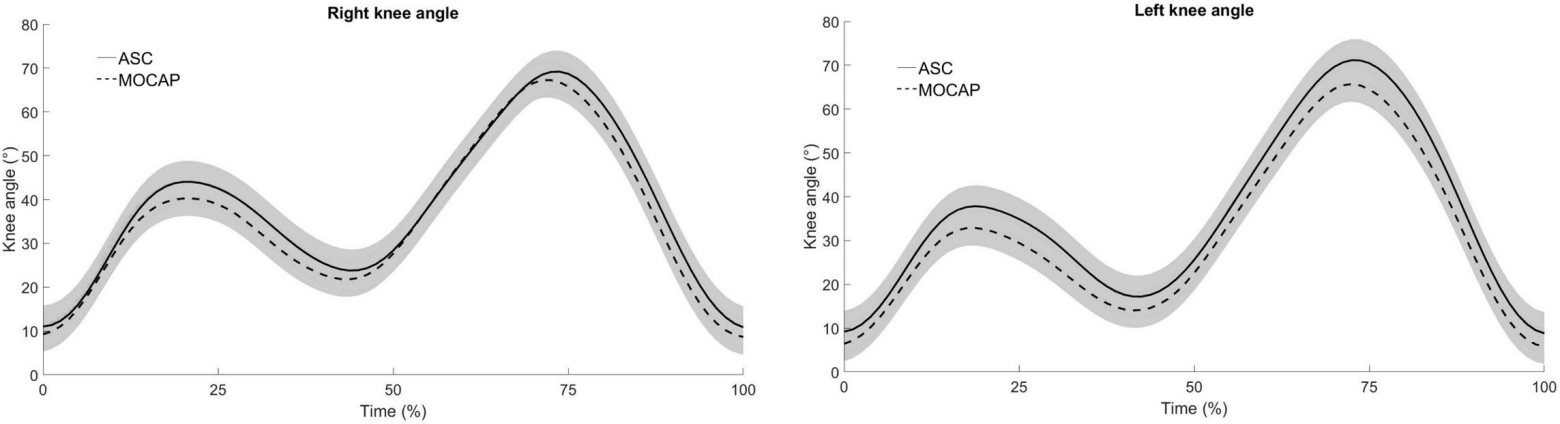
Mean curve of the angular variation, of the right and left knee, of 14 stride cycles in one running trial (40 meters). Vicon (dashed line) and GoPro (continuous line).

The left and right knee angles showed a similar pattern, referring to a comparison between the systems. The waveform of the knee flexion-extension angle in each gait cycle, obtained by ASC and MOCAP systems, in the setup #3, were highly correlated in the walking (right lower limb: *ρ*>0.96, p<0.05; left lower limb: *ρ*>0.94, p<0.05) and in the running (right lower limb: *ρ*>0.97, p<0.05; left lower limb: *ρ*>0.98, p<0.05). The curve patterns as a function of the stride cycle can be checked on the Fig 2 (walking) and Fig 3 (running).

The angular and linear parameters of the walking and the running, performed in the setup #3, were reported in the Table 4. In relation to the walking task were not found significant differences between both systems in the variables calculated for each cycle (p>0.05; effect size: r = 0.01 and r = 0.01 to the right and left stride length, respectively; r = 0.11 and r = 0.10 to the maximum values of the right and left knee angle, respectively). The same results were found to the running task, without significant differences for the stride length (*p*>0.05; effect size: r = 0.01 and r = 0.01 to the right and left, respectively) and for the maximum knee angle (*p*>0.05; effect size: r = 0.01 and r = 0.42 to the right and left, respectively). The average velocity in which the subject performed the walking task was similar for the two systems. For the mean velocity in the running task we also found similar values (Table 4).

**Table 4:** Mean results (± standard deviation) of the walking and running parameters, calculated for both limbs, obtained by ASC and MOCAP: Stride length, right and left maximum knee angle, mean velocity and the difference between the values of both systems (Δ).

Walking parameters*										
	**Stride length** **[m]**				**Maximum Knee Angle** **[degrees]**				**Mean Velocity [m/s]**	
	**Right**		**Left**		**Right**		**Left**			
	**ASC**	**MOCAP**	**ASC**	**MOCAP**	**ASC**	**MOCAP**	**ASC**	**MOCAP**	**ASC**	**MOCAP**
Setup #1	1.01±0.06	1.02±0.06	1.17±0.05	1.18±0.08	70.87±2.02	68.60±2.08	63.93±3.41	62.37±3.25	0.63±0.05	0.64±0.05
Δ	-0.015		-0.01		2.3		1.6		-0.03	
***p-value***	0.700		0.999		0.400		0.700		-	
Setup #3	1.21±0.04	1.21±0.04	1.21±0.05	1.20±0.05	62.71 ±2.12	62.37 ±2.06	57.57 ±1.15	57.01 ±1.79	0.75	0.89
Δ	-0.003		0.0003		0.3379		0.5549		-0.1418	
***p-value***	0.973		0.962		0.535		0.667		-	
Running parameters*										
	**Stride length** **[m]**				**Maximum Knee Angle** **[degrees]**				**Mean Velocity [m/s]**	
	**Right**		**Left**		**Right**		**Left**			
	**ASC**	**MOCAP**	**ASC**	**MOCAP**	**ASC**	**MOCAP**	**ASC**	**MOCAP**	**ASC**	**MOCAP**
Setup #3	1.32±0.2	1.32±0.2	1.32±0.2	1.32±0.2	68.88±4.80	67.95 ±4.08	70.87 ±3.76	68.57 ±3.44	1.56	1.58
Δ	-0.001		-0.07		0.93		2.31		-0.02	
***p-value***	0.945		0.945		0.982		0.077		-	

m = meters; m/s = meters per second; ASC = Action Sport Camera; MOCAP = Motion Capture System; Δ = difference between the values of ASC and MOCAP system; *p*-value = comparison of the walking and running parameters between ASC and MOCAP, p<0.05.

*Mean values of cycles.

## Discussion and conclusion

In this study, walking and running parameters reconstructed by an optoelectronic motion capture (MOCAP) system were compared to the corresponding parameters obtained by adopting a customized system based on commercial action sport cameras (ASC) in different acquisition setups. This aim was motivated by the need of addressing some issues of the traditional acquisition setups in biomechanics laboratory, which are expensive, require cables and infrared illuminators and survey only a limited working volume. In particular, the originality of this work was to propose a system using action sport cameras based on the moving cameras procedure to follow the displacement of the subject allowing a less constrained movement compared to the laboratory setup.

In general, the MAE values were smaller than 2.6mm and 1.3 degrees for ASC and less than 0.7mm and 0.6 degrees for MOCAP. This study confirms the small reconstruction errors for the two systems, previously investigated [16,17,1]. The statistical analysis confirms also bigger MAE values in the ASC than MOCAP. To a certain extent, this result was expected since the optical systems are considered by the literature as gold standard systems for the capture of movement [21]. However, although the absolute accuracy on a rigid bar was more reliable in the MOCAP system, with a mean absolute error ranging from 2.5 to 15 times lower than the ASC for all the variables evaluated, when we analyze the percentage errors, the ASC presented relative errors for D1, D2 and angle less than 2%. These small instrumental errors may not directly affect the analysis of movements with larger amplitudes, as for example the gait, with scale in meters.

We hypothesized that the two systems could present a decreased accuracy results because of volume extension provided by the mobile structure. This was built on the fact that the MOCAP was designed to be used in a controlled environment and they are sensitive to external lights and camera disturbances [21]. In the case of ASC, designed to be used in different conditions and that present low reconstruction errors (< 3 mm) [14,15,1], they depend on good contrast maintenance in the image between markers and background.

Our results confirmed that the MOCAP is sensitive to non-laboratory conditions, since we found worst MAE values in setup #2 and #3. This was evidenced by an increase, of almost 3 times, in error in D2, in comparison of the setup #1 and #2 [MAE D2 setup #2 / MAE D2 setup #1] with the environment variation. Instead, no effects were found in the other distance evaluated with the environment variation. This fact may be linked to the instability of the system when placed in a non-laboratory condition. It seems unable to maintain the distortion corrections, and this may have influenced in one distance more than the other. On the other hand, in the ASC system evaluation, this first hypothesis was not confirmed. The inter-markers length D1 and D2 did not change their relation [MAE values setup #2 / MAE values setup #1], that were approximately equal 1 in both distances, suggesting that the errors remained the same. In contrast, we noticed, observing the images, that the reconstructions performed by ASC were more affected by the calibration quality. The D1 values are 1.5 times greater than D2 values (Table 3). This may be associated with the rigid bar movement across the whole acquisition volume, from the middle of the image to the edges, where the radial distortion has more influence. As found in the literature, the reconstruction accuracy results can be affected by the wand calibration movement [16,22]. A calibration movement that spreads more the acquisition volume may allow minors reconstruction errors. In both systems, a change in the reconstruction error values of the inter-markers distances, even if not significantly as occurred in the ASC, reflected on the angle values of the rigid bar. Although the value was very close to the real value (90°), with errors below 1.2 degrees (Table 3), both systems presented statistical differences in the evaluation of the angle as a function of time in the setup #2 compared to the laboratory setup.

The second hypothesis was that both systems could have decreased accuracy results with the moving cameras (setup #3), however, when describing walking and running movements, the two systems would not have differences in the computed kinematic variables. This hypothesis was also confirmed by our results. One fact that may have influenced the two systems was a possible camera position change during the acquisition of the trials, since moving the mobile structure can cause vibrations that can affect the accuracy of the 3D marker reconstruction. A decreasing in the accuracy results was already reported using the MOCAP system in this condition [13]. Even performing the camera calibration based on procedure [13], it was not enough to correct the possible camera vibrations affecting the intrinsic and extrinsic camera parameters and consequently, the reconstruction precision. Thus, the two systems appear to have been affected by this in a similar way.

Particularly in the ASC (setup #3), the inter-markers length D1 decreases the reconstruction error. As discussed previously, the edges of the image present greater radial distortion. In this setup, the structure is moving the cameras and the movement of the rigid bar is concentrated in the middle of the volume. Thus, the radial distortion, coupled with a better calibration seems to have been better corrected, and thus, a smaller reconstruction error was found.

The significant differences between the accuracy results between each experimental condition (setups) suggest that the system’s accuracy in setups outside the lab cannot be extrapolated based on evaluations in the lab condition, and the same should be considered for the conditions of the moving cameras.

Despite of these evaluation results about the system’s performance, in the human movement parameters, walking and running, no significant differences were found between the systems (Table 4). We can point out that the pattern of the right-left knee angles is very similar between MOCAP and ASC (Fig 2 - walking and Fig 3 - running).

One of the contributions of this study was to show the possibility of acquiring larger quantities of the movement cycles. As mentioned in the methods, the usage of a mobile structure setup allowed the acquisition of 17 cycles in the walking trial, accounting 34 maximum knee angle values and 34 stride length values (17 cycles x 2 lower limbs), and 14 cycles in the running trial, accounting 28 angle values and 28 stride length values (14 cycles x 2 lower limbs). This can mainly contribute to the definition of the movement patterns or a better ecological movement description. The studies that evaluate the gait, whether in healthy subjects or in a group that presents some pathology, records an average of 3 to 6 stride cycles per acquisition [2,3]. Thus, in these cases, in order to define a movement pattern it is necessary several analyzes of this same movement. When the movements are cyclical, evaluating few cycles at each acquisition and then merging the data can be considered an erroneous way of defining a pattern.

Considering that it was the same subject evaluated in the setup #1 and #3 and that the velocity was self-selected, the mobile structure seems to have not interfered in the gait pattern of the volunteer. We could notice this from the linear and angular gait values found between the laboratory environment (setup #1) and outside the laboratory (setup #3) presented close values. Despite of this, to acquire large amounts of cycles of the movement through the ASC is a question that should be considered. It was possible to notice that, with the mobile structure, there is a better distribution of the stride length values between the lower limbs, wherein the setup #1 there is a tendency of the left stride to be longer than the right one. In the setup #3, the values between the two lower limbs resemble each other (Table 4). Thus, although the volunteer does not have to bother about the capture system in a laboratory controlled acquisition, this condition may interfere with this variable. Further investigation with more subjects is needed to confirm whether this behavior holds.

Forwarding to the end, some limits of the present study must be discussed. First, we can point out that a limitation of this study is related to minimizing the markers occlusion. We used a marking protocol that involved markers on both lower limbs and pelvis and a setup with only four cameras, a minimum number for bilateral body evaluation. Therefore, including more cameras could reduce these losses or occlusions [23].

Second, to create an automated system to move the structure is an issue that we want to address in future studies to avoid interferences on the act of pushing the mobile structure. However, before considering this it is necessary to deepen the tests with the ASC under new conditions, as for example at other movement speeds. Our results do not show that the mobile structure cannot disturb the subject’s motion, however higher movement speeds could better identify if whether or not the structure actually interferes in the movement.

Third, the study was limited to angular measurements in the sagittal plane to the human kinematic data. Although the frontal and transversal plane motions are one of the concerns in sports biomechanics, they are more prone to error even in traditional motion capture setups. Therefore, we use the most common variables to describe the tasks, because in order to report more complex and full body 3D kinematic data it is necessary to explore the ASCs as to their validity in this context of the moving cameras.

It is necessary to improve the analyses using ASC, however these results present an initial evaluation on the 3D reconstruction using this type of camera, in motion. The ASC offers advantages in terms of absence of cables, which makes the setup process easy to get them moving. In future research, besides improving acquisition protocols and deepen the accuracy evaluation, it would be interesting, to use more cameras in order to minimize the limitations found in this study, as well as more in-depth evaluations of human movement in an attempt to identify gait dysfunctions or to define a pattern, for example.

## Supporting information

S1 File Code of calibration. (ZIP)

S2 File 3D reconstruction accuracy—Rigid bar test with the action sport cameras in setup #1. (TXT)

S3 File 3D reconstruction accuracy—Rigid bar test with the optoelectronic system in setup #1. (C3D)

S4 File 3D reconstruction accuracy—Rigid bar test with the action sport cameras in setup #2. (TXT)

S5 File 3D reconstruction accuracy—Rigid bar test with the optoelectronic system in setup #2. (C3D)

S6 File 3D reconstruction accuracy—Rigid bar test with the action sport cameras in setup #3. (TXT)

S7 File 3D reconstruction accuracy—Rigid bar test with the optoelectronic system in setup #3. (C3D)

S8 File Walking data with the action sport cameras in setup #1. (TXT)

S9 File Walking data with the optoelectronic system in setup #1. (C3D)

S10 File Walking data with the action sport cameras in setup #3. (TXT)

S11 File Walking data with the optoelectronic system in setup #3. (C3D)

S12 File The overlapping 3D coordinates of the walking data in the setup #3, reconstructed by the two systems. (C3D)

S13 File Running data with the action sport cameras in setup #3. (TXT)

S14 File The overlapping 3D coordinates of the running data in the setup #3, reconstructed by the two systems. (C3D)
